# Accuracy of a novel accelerometer-based navigation (Naviswiss) for total hip arthroplasty in the supine position

**DOI:** 10.1186/s12891-022-05495-3

**Published:** 2022-06-04

**Authors:** Masahiro Hasegawa, Yohei Naito, Shine Tone, Akihiro Sudo

**Affiliations:** Department of Orthopaedic Surgery, Mie University Graduate School of Medicine, 2-174 Edobashi, Tsu City, Mie 514-8507 Japan

**Keywords:** Total hip arthroplasty, Navigation, Acetabular cup, Accelerometer, Supine position

## Abstract

**Background:**

This study aimed to determine the accuracy of acetabular cup insertion using a novel accelerometer-based navigation system in total hip arthroplasty (THA).

**Methods:**

A single-surgeon study was conducted in which 62 prospective patients with navigation and 42 retrospective patients without navigation in a supine position were compared. Absolute values for errors of radiographic inclination and anteversion were calculated. Navigation error was also calculated. Factors that affected absolute value of navigation error in cup alignment were determined.

**Results:**

In the navigation group, mean absolute errors for radiographic inclination and anteversion were 4.1° and 4.3°, respectively. In the control group, mean absolute errors were 6.6° in inclination (*p* < 0.01) and 5.9° in anteversion (*p* = 0.04). Mean absolute values of navigation error were 2.8° in inclination and 2.8°in anteversion. Factors affecting navigation errors were not found.

**Conclusion:**

This novel accelerometer-based navigation system significantly increased the accuracy of cup placement during THA in the supine position.

## Background

Accurate cup positioning in total hip arthroplasty (THA) is important to prevent impingement [1], dislocation [2], wear, and loosening [3]. To reduce the rate of dislocation, anterior and anterolateral approaches are reportedly useful [4]. Placement with navigation is more precise, with improved accuracy for cup inclination and anteversion compared to conventional placement [5]. However, such large console navigation systems are not popular because of the high cost. The costs are much lower for portable navigation systems than for large console navigation systems. An accelerometer-based portable navigation system (HipAlign; OrthAlign, Aliso Viejo, CA) has been used in THA, comprising accelerometers, gyroscopes, and inertial detectors to communicate between a reference sensor and the display unit [6–13]. Several studies have reported that HipAlign has significantly improved the accuracy of cup inclination and anteversion [6–13].

**Fig. 1:**
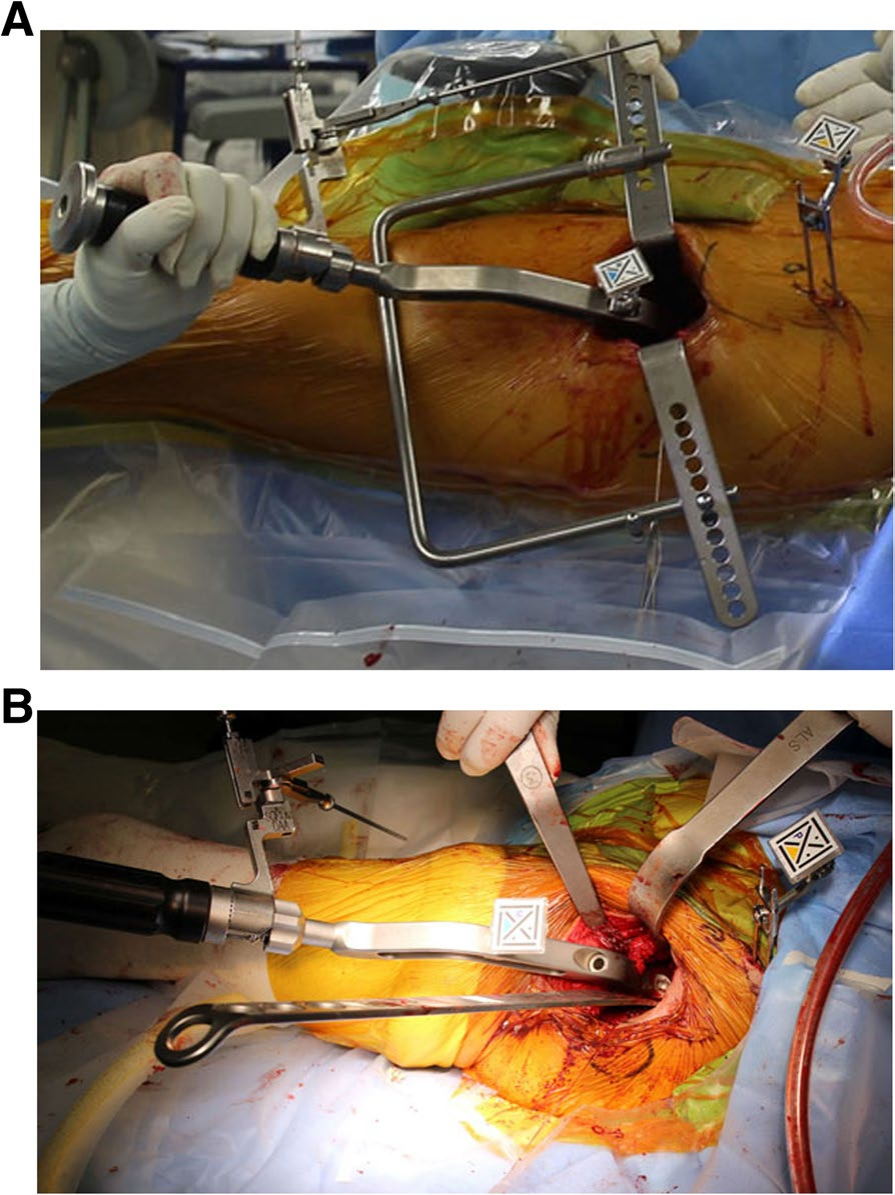
**A** Cup insertion with the direct anterior approach (DAA). **B** Cup insertion with the anterolateral supine approach (ALS). Navigation tags are attached to fixation pins at the pelvis and cup inserter

**Fig. 2:**
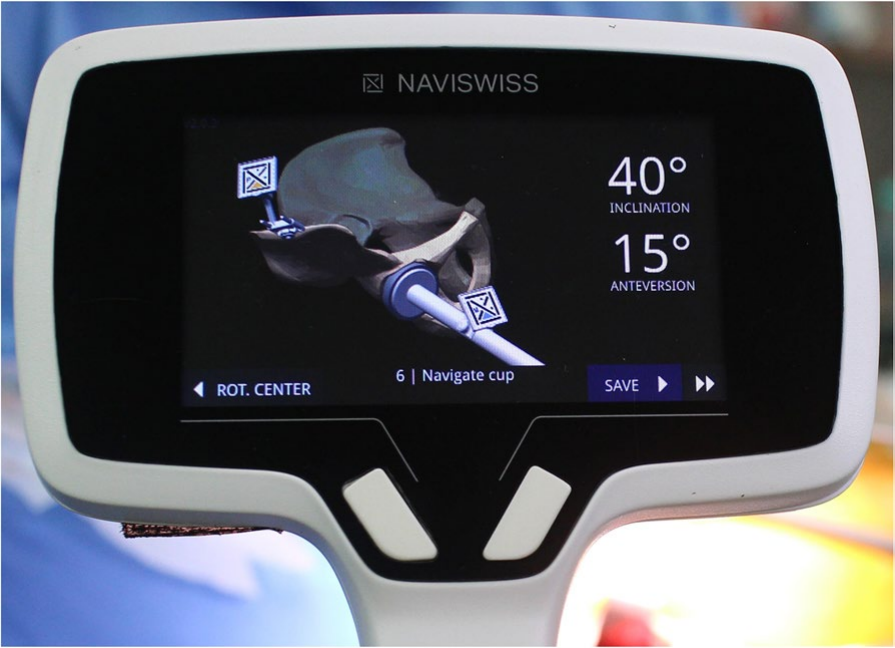
The Naviswiss monitor after cup insertion showing 40° of inclination and 15° of anteversion

The Naviswiss system (Naviswiss AG, Brugg, Switzerland) is a novel portable navigation system, comprising a handheld navigation device. The navigation unit includes an infrared stereo camera that measures the position and orientation of a small tag mounted to the pelvis. The unit attached to the cup impactor is also small and lightweight with only a tag (Figs. 1, 2). An infrared flash illuminates the field of view to avoid disturbances in available light. An inertial measurement unit (IMU) with accelerometers and gyroscopes is built-in to measure the camera orientation in space. Pelvic calipers are used to identify the anterior superior iliac spine (ASIS) bilaterally to establish the functional pelvic plane (FPP), combined with IMU data to establish the gravitational axis and embed a coordinate system into the pelvis during the procedure [14].

The hypothesis was that use of Naviswiss in a supine position is more precise than the conventional technique for acetabular cup placement in THA.

## Materials and methods

### Patients

Between April 2020 and May 2021, a total of 62 consecutive, prospectively enrolled patients underwent primary THA in a supine position using Naviswiss under general anesthesia. All procedures were performed by the same surgeon (M.H.). The hip was exposed via a direct anterior approach (DAA, Fig. 1A) on a traction table [15, 16] or with a modified Watson-Jones approach (anterolateral supine approach [ALS], Fig. 1B). Fifty-five hips were treating using the DAA, with the remaining 7 hips treated using the ALS. The ALS was selected in cases of severe deformity or excessive anteversion of the femoral neck. A Squrum TT Shell (Kyocera, Kyoto, Japan) was used in all patients treated using Naviswiss.

As a control group, 42 previously reported patients who had undergone THA via an ALS approach in the supine position between June 2015 and June 2017 were included [8]. A G7 PPS Finned BoneMaster Acetabular Shell (Zimmer Biomet, Warsaw, IN) had been used in each of the control group. These surgeries also performed by the same surgeon (M.H.). Demographic characteristics of patients are shown in Table 1.

**Table 1:** Patients’ demographic characteristics

			Navigation group	Control group	*p*-value
Age^a^	(yrs)		68 ± 10	66 ± 11	0.242
Sex	Male		6	9	0.165
	Female		56	33	
BMI^a^	(kg/m2)		24.6 ± 4.9	24.0 ± 3.8	0.799
Diagnosis	OA		60	38	0.218
	Crowe group	1	56	38	0.155
		2	4	0	
	ONFH		2	4	

*BMI* Body mass index, *OA* Osteoarthritis, *ONFH* Osteonecrosis of the femoral head

^a^Values are given as mean ± standard deviation

Two fixation pins (diameter, 3.0 mm) were placed on the ipsilateral iliac crest to fix a single-use sterilized tag (P-tag) in the navigation group (Fig. 1). The FPP using Naviswiss was determined with the simultaneous palpation of both ASISs using pelvic calipers with M-tag. The built-in gravity sensor automatically referenced the horizontal operation table level. Assistant held the navigation unit including camera, and moved the unit to view both tags (P-tag attached to pelvis and M-tag attached to the cup impactor) during cup impaction. After viewing both tags, inclination and anteversion angles were shown on the monitor (Fig. 2). The report was exported directly to USB memory for further storage or printout. Press-fit fixation could be obtained in all cases after 1-mm under-reaming without use of screws. The target angle of cup radiographic inclination was 40°. Cup radiographic anteversion was targeted to 15° relative to the FPP in the hips with navigation. In the control group, the target angle of radiographic inclination was 40° relative to horizontal line defined by bilateral ASISs, however, radiographic anteversion was targeted to 20° relative to the operation table, because we used a mechanical guide for 20° radiographic anteversion [8].

For all analyses, radiographic angles were used based on the definitions by Murray [17]. Radiographic inclination and anteversion angles of the cup, relative to the FPP, were displayed on the monitor (Fig. 2). We checked inclination and anteversion angles before impaction of the cup, and performed adjusted to aiming angles. The final inclination and anteversion angles were re-checked after cup impaction, and these angles were used for calculation of errors.

### Evaluation

Computed tomography (CT) was performed from the pelvis to the knee joint at 2 weeks postoperatively. Component positions were measured postoperatively by uploading 3-dimensional digital imaging and communications in medicine (DICOM) data to dedicated software (ZedHip; Lexi, Tokyo, Japan). Cup inclination and anteversion angles were measured with respect to the FPP by one observer (Y.N.). Intra- and inter-observer reliabilities in this measurement have been studied previously. Intra-observer reliabilities were 0.915 and 0.963 in inclination and anteversion, respectively. Inter-observer reliabilities were 0.951 and 0.937 in inclination and anteversion, respectively [8].

Absolute values of errors in radiographic inclination and anteversion with respect to the FPP were calculated by subtracting preoperative target angles from the angles of postoperative CT measurement. Intraoperative inclination and anteversion angles using navigation were recorded. Navigation error was defined and was calculated by subtracting angles of the intraoperative navigation record from the angles of postoperative CT measurement [8]. Percentages of hips with navigation errors over 5° and 10° were determined. Percentages of the hips inside the “safe zone”, as detailed by Lewinnek et al. [2] (Lewinnek safe zone: inclination between 30° and 50°, anteversion between 15° and 35°) were compared. All patients were followed after THA, and complications and dislocation were examined. All methods were performed in accordance with the Declaration of Helsinki. Our institutional review board approved this study (H2018–083).

### Statistical analysis

From a previous portable navigation study [7], difference (mean ± standard deviation) in navigation and conventional groups for cup inclination was 2.8° ± 2.4°. Based on this finding, a sample size of 12 hips in each group was considered necessary to detect a significant difference between groups (ɑ = 0.05, power = 0.8). The demographic characteristics of patients, including age and body mass index (BMI) were compared between groups using the Mann-Whitney U-test. The chi-squared test and Fisher’s exact test were used to compare sex, diagnosis, and Crowe group. The Mann Whitney U-test was used to determine differences (errors) between intraoperative target angles and postoperative CT measurements between groups. Fisher’s exact test was used to compare cups inside the “safe zone”, as detailed by Lewinnek et al. [2] (Lewinnek safe zone).

Factors that affected the absolute value of navigation error in cup alignment in the navigation group were determined. The Mann-Whitney U-test was used to determine differences in terms of sex, preoperative diagnosis, Crowe group, and approach. Correlation analyses were performed using Spearman’s rank correlation test. In these analyses, dependent variables included age, BMI, and preoperative pelvic tilt. Values of *p* < 0.05 were considered significant. IBM SPSS Statistics version 24 (IBM Corp., Armonk, NY) was used for all analyses.

## Results

**Fig. 3:**
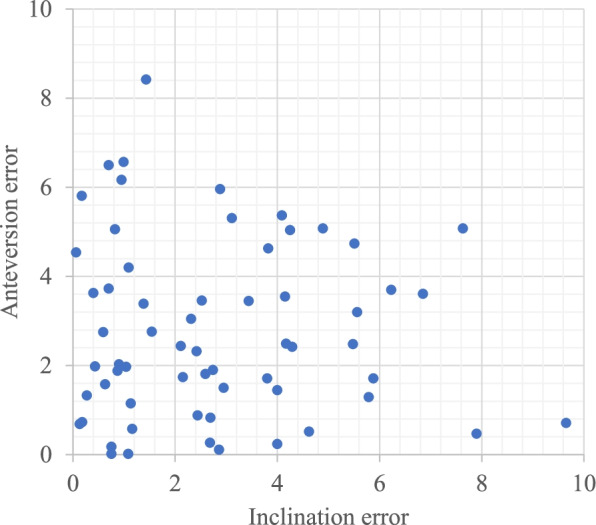
Scatter plot of absolute value of navigation error in radiographic inclination and anteversion

No significant differences in demographic characteristics were identified between groups (Table 1). In the navigation group, mean postoperative radiographic inclination and anteversion relative to the FPP were 36.5° ± 3.9° (range, 26.4–46.6°) and 17.7° ± 4.6° (range, 8.8–29.4°), respectively. Mean absolute errors in postoperatively measured angles from target angles for radiographic inclination and anteversion (postoperative CT measurement - target angle) were 4.1° ± 3.2° and 4.3° ± 3.2°, respectively. In the control group, mean postoperative radiographic inclination and anteversion relative to the FPP were 34.6° ± 5.8° (range, 20.9–48.1°) and 21.4° ± 7.0° (range, 7.2–39.5°), respectively. Mean absolute errors of postoperatively measured angles from the target angles for radiographic inclination and anteversion were 6.6° ± 4.4° and 5.9° ± 4.0°, respectively [8]. The navigation group showed better results for mean absolute errors in postoperatively measured angles from the target angles in both inclination (*p* = 0.002) and anteversion (*p* = 0.044). Mean absolute values for navigation error (postoperative CT-navigation record) were 2.8° ± 2.2° (range, 0.1–9.7°) in inclination and 2.8° ± 2.0° (range, 0.0–8.4°) in anteversion (Fig. 3).

Percentages of hips with navigation errors > 5° were 15% in radiographic inclination and 10% in radiographic anteversion. No hips showed navigation errors > 10°. No factors significantly affecting absolute value of navigation error were found for radiographic inclination or anteversion (Table 2). Percentages of cups inside the Lewinnek safe zone were 95.2% in the navigation group and 66.7% in control group (*p* < 0.001). No complications arising at pin sites were seen, and no dislocations occurred in any hips.

**Table 2:** Factors affected the absolute value of navigation error of cup position

	Inclination		Anteversion	
	*p*-value	r	*p*-value	r
Sex	0.924		0.896	
Diagnosis	0.690		0.486	
Crowe group	0.465		0.699	
Approach	0.815		0.609	
Age	0.915	−0.014	0.360	−0.118
BMI	0.496	0.088	0.409	0.107
Preoperative pelvic tilt	0.508	0.086	0.247	−0.149

*BMI* Body mass index

## Discussion

The present study is the first, to the best of our knowledge, to demonstrate the accuracy of cup placement using the Naviswiss system during THA. In CT-based navigation, absolute values of navigation error were reported in the range of 1.2–4.6° in inclination, and 1.0° to 4.4° in anteversion [18–23]. The absolute deviations of the postoperative measured angle from the target position were ranged from 1.5° to 4.2° in inclination, and 1.6° to 5.3° in anteversion [18, 23]. In image-free large-console navigation, absolute values of navigation error in inclination reportedly ranged from 2.1° to 4.4°, and anteversion errors ranged from 3.7° to 7.1° (Table 3) [18, 20, 24–26]. The absolute deviations of the postoperative measured angle from the target position were ranged from 2.8° to 3.6° in inclination, and 4.2° to 5.1° in anteversion [18, 24, 26]. Anteversion with CT-based navigation systems has been reported to offer superior accuracy compared to imageless large-console navigation systems. Anteversion with image-free navigation is based on the APP, which does not contain the pelvic tilt angle. Percutaneous palpation of the pubis is quite imprecise [27], and the thickness of soft tissue overlying the pubic symphysis affects the accuracy in anteversion. Accurate registration of the APP is required to achieve greater consistency in cup placement with image-free large-console navigation. Davis et al. [28] reported that the ipsilateral ASIS and spinous process of the L5 vertebra or bilateral ASIS points were registered without registration of the pubis. However, the former registration was reportedly inferior to the latter registration [20]. CT-based navigation demonstrated excellent accuracy in cup placement, especially with severe deformities such as Crowe type IV [23]. Using accelerometer-based navigation (HipAlign), absolute values of navigation error in inclination reportedly ranged from 2.6° to 3.7°, and navigation errors in anteversion ranged from 2.7° to 3.0° in the supine position. The absolute deviations of the postoperative measured angle from the target position were ranged from 2.6° to 3.8° in inclination, and the values in anteversion were ranged from 2.7° to 3.8° [6–10]. The absolute value of navigation error in inclination was reported as 3.2° and navigation error in anteversion was 6.0° in the lateral decubitus position [13]. The absolute deviation of the postoperative measured angle from the target position was 3.7°in inclination, and the values in anteversion were ranged from 5.9° to 6.0° [11, 12]. HipAlign of the supine position seemed to be better for anteversion accuracy compared with that of the lateral decubitus position. When HipAlign is used in the supine position, bilateral ASISs are registered, and FPP is determined by sensing the gravitational axis. Determining FPP working with accelerometers and gyroscopes is one of the benefits of supine THA using HipAlign. When used HipAlign in the lateral decubitus position, the longitudinal plane of body is registered by holding the registration probe parallel to long axis of body [11]. Pelvic position fixed by the positioner could be the major factor affecting the anteversion accuracy in the lateral decubitus position [13].

**Table 3:** Accuracy of navigation systems

Navigation	Authors	Company	Position	Absolute value of error^e^		Absolute value of navigation error^f^	
				Inclination (°)	Anteversion (°)	Inclination (°)	Anteversion (°)
CT-based	Kalteis et al. [18]	Brainlab	Supine	4.2 ± 4.0	5.3 ± 5.3	3.0 ± 2.6	3.3 ± 2.3
	Yamada et al. [19]	Brainlab^a^	Lateral			2.5 ± 2.2	2.3 ± 1.7
	Yamada et al. [19]	Brainlab^b^	Lateral			4.6 ± 3.3	4.4 ± 3.3
	Hasegawa et al. [20]	Brainlab^a^	Lateral			1.9 ± 1.5	3.0 ± 2.3
	Iwana et al. [21]	Stryker	Lateral			1.8 ± 1.6	1.2 ± 1.1
	Nakahara et al. [22]	Stryker	Lateral			1.2 ± 1.3	1.0 ± 0.8
	Ueoka et al. [23]	Stryker	Lateral	1.5 ± 1.1	1.6 ± 1.2	1.2 ± 1.0	1.4 ± 1.0
Image-free	Tsukada and Wakui [24]	B. Braun Aesculap	Supine	2.8 ± 2.5	4.2 ± 3.0	2.4 ± 2.0	3.7 ± 2.3
	Fukunishi et al. [25]	B. Braun Aesculap	Supine			3.0 ± 2.6	5.0 ± 3.5
	Kalteis et al. [18]	Brainlab	Supine	3.6 ± 4.0	4.2 ± 5.5	2.9 ± 2.2	4.2 ± 3.3
	Hasegawa et al. [20]	Brainlab^c^	Lateral			4.4 ± 4.2	7.1 ± 6.3
	Hasegawa et al. [20]	Brainlab^d^	Lateral			2.1 ± 1.8	4.1 ± 3.6
	Naito et al. [26]	Brainlab^d^	Supine, Lateral	3.4 ± 3.0	5.1 ± 3.6	3.3 ± 2.8	5.8 ± 4.9
Accerelometer-based	Kamenaga et al. [6]	OrthAlign	Supine			2.6 ± 2.7	2.8 ± 2.7
	Takada et al. [7]	OrthAlign	Supine	3.3 ± 2.7	3.8 ± 3.4		
	Hasegawa et al. [8]	OrthAlign	Supine	3.8 ± 2.7	3.3 ± 2.5	3.7 ± 2.8	3.0 ± 2.6
	Hayashi et al. [9]	OrthAlign	Supine	2.6 ± 1.9	2.7 ± 2.2	2.7 ± 2.1	2.7 ± 1.8
	Okamoto et al. [10]	OrthAlign	Supine	3.1 ± 2.2	2.8 ± 2.3		
	Tanino et al. [11]	OrthAlign	Lateral	3.7 ± 3.0	6.0 ± 4.5		
	Tanino et al. [12]	OrthAlign	Lateral	3.7 ± 3.3	5.9 ± 3.6		
	Tsukamoto et al. [13]	OrthAlign	Lateral			3.2 ± 2.2	6.0 ± 4.1
	Present study	Naviswiss AG	Supine	4.1 ± 3.2	4.3 ± 3.2	2.8 ± 2.2	2.8 ± 2.0

Absolute errors are given as means ± standard deviation

^a^CT-based 2-dimensional to 3-dimensional matched navigation

^b^paired-point matched navigation

^c^ipsilateral ASIS and L5 spinous process registration

^d^bilateral ASIS registration

^e^absolute deviation of the postoperative measured angle from the target position

^f^absolute difference between the navigation recorded and the postoperative measured angle

Most cups were placed inside the Lewinnek safe zone in the navigation group. Many studies have questioned the utility of this so-called “safe zone” [29–31]. Drawing broad conclusions regarding the definitive target zone for cup placement is difficult due to the likely multifactorial nature of dislocation after THA [30].

One of the most useful points of Naviswiss with the patient in the supine position is that the surgeon can determine cup angles relative to the FPP working with accelerometers and gyroscopes, like HipAlign (OrthAlign). Absolute values of errors using HipAlign in the supine position are similar to those reported in the present study [6–10]. One of the reasons might be that cup angles relative to the FPP working with accelerometers and gyroscopes could be provided in both systems in the supine position. The advantages of using the Naviswiss included the fact that the diameter of fixation pins on the iliac crest were smaller (3.0 mm), resulting in reduced invasiveness in the iliac crest. In HipAlign, the reference sensor is needed to attach to the cup impactor from the metal pelvic base for checking cup angles. Display time of cup angles on the navigation unit is 30 seconds after registration, and surgeons are often required to repeat the registration step several times. In Naviswiss, surgeons can check inclination and anteversion angles during impaction without repeat registration. When Naviswiss is used in the lateral decubitus position, surgeons can change in patient position to the lateral decubitus position after determining the FPP in the supine position. The method for using Naviswiss in the lateral decubitus position might be a great advantage for improving anteversion accuracy.

The following factors may affect the accuracy of portable navigation system in the supine position. First, there is a limitation in the verification method. The pelvic tilt on the operating table could be different from the pelvic tilt at CT imaging. Second, registration errors could occur to palpate ASIS percutaneously. Third, loosening of fixation pin on the iliac crest could occur during surgery. Fourth, supplemental screw fixation might move the cup position. In the present study, supplemental screw fixation was not used. Press fit without screws was also an advantage in the present study to minimize evaluations for navigation error.

The present study showed several limitations. First, only a small number of patients was studied. Second, the navigation group included both DAA and ALS approaches, although the control group included only the ALS approach. Third, cases with severe deformities such as Crowe type IV were not evaluated. Fourth, anteversion errors might occur if the pelvis remained rotated during surgery in the control group [16]. Fifth, clinical results were lacking. Further studies are needed to show the clinical advantages of the Naviswiss.

## Conclusions

A novel navigation system (Naviswiss) significantly improved the accuracy of cup placement when compared to freehand placement during THA in a supine position, and our hypothesis as verified. Using the Naviswiss, mean errors were 2.8° in inclination and 2.8° in anteversion.

## Data Availability

The datasets during and/or analyzed during the current study are available from the corresponding author on reasonable request.

## Abbreviations

THA: Total hip arthroplasty
IMU: Inertial measurement unit
ASIS: Anterior superior iliac spine
FPP: Functional pelvic plane
DAA: Direct anterior approach
CT: Computed tomography
DICOM: Digital imaging and communications in medicine
BMI: Body mass index
